# Safety and efficacy of autologous adipose-derived stem cells for knee osteoarthritis in the elderly population: A systematic review

**DOI:** 10.1016/j.jcot.2024.102804

**Published:** 2024-11-07

**Authors:** Biagio Zampogna, Francesco Rosario Parisi, Augusto Ferrini, Andrea Zampoli, Giuseppe Francesco Papalia, Saseendar Shanmugasundaram, Rocco Papalia

**Affiliations:** a Department of Orthopaedic and Trauma Surgery, Università Campus Bio-Medico di Roma, Via Alvaro del Portillo, 21, 00128, Roma, Italy; b Research Unit of Orthopaedic and Trauma Surgery, Fondazione Policlinico Universitario Campus Bio-Medico, Via Alvaro del Portillo, 200, 00128, Roma, Italy; c BIOMORF Department, Biomedical, Dental and Morphological and Functional Images, University of Messina. A.O.U. Policlinico “G. Martino”, Messina, Italy; d Sri Lakshmi Narayana Institute of Medical Sciences, Puducherry, India

**Keywords:** Adipose-derived stem cells, Cartilage, Knee osteoarthritis, Regenerate, Elderly

## Abstract

**Introduction:**

Osteoarthritis (OA) is a progressive joint disease, and over 240 million people suffer from symptomatic OA, primarily in the knee, and mainly affects the elderly population over 65. A combination of different risk factors leads to biological changes in the microenvironments of the joints, causing cartilage overload and chondrocyte aging. Adipose-derived MSCs (ADSCs) are demonstrated to improve joint environments with an effective therapy for Knee OA. This review focused on patients over 65 years old to evaluate the effectiveness of ADSC therapies in treating KOA in elderly patients and demonstrate that complications are not higher in this cohort of patients.

**Materials and methods:**

We conducted a bibliography search through the PubMed, Scopus, and Cochrane databases for English-language and human clinical trials published until Feb 7, 2024. We extracted the following study characteristics: Authors, year of publication, type of study, number of patients, number of knees, sex, Kellgren-Lawrence classification, culture ADSC, Number of cells injected, mean follow-up, adverse events, significant complications, and clinical outcomes data were extracted recorded and analyzed.

**Results:**

According to inclusion criteria, seven clinical trials on autologous adipose-derived stem cells were considered. Four studies analyzed stem cells as a stromal vascular fraction (SVF), two as ADSC cultured, and 1 study investigated the MAT procedure. All studies reported improved clinical outcomes using autologous adipose-derived stem cells, on 339 knees. Post-treatment increased KOOS, WOMAC, IKS, VAS, and Lysholm knee scores were highlighted. All studies showed an improvement in all outcomes scores, and regarding complications, only 44 knees underwent adverse events, but no significant complications were found in all the studies reported.

**Conclusions:**

The current systematic review demonstrated that using autologous adipose-derived stem cells improved clinical outcomes and is effective and safe in elderly patients. Additionally, this study will encourage orthopedic surgeons not to consider surgery as the only solution in elderly patients who are refractory to treatment and do not show end-stage knee osteoarthritis.

**Level of evidence:**

Level IV, systematic review of level IV studies**.**

## 1 Introduction

Osteoarthritis (OA) is a progressive joint disease that disrupts the anatomy and the biomechanics of the joint.^1^ Several studies clearly have explained the increasing trends of OA^2^ because it represents one of the highly worldwide prevalent chronic pain diseases that leads to disability and loss of function.^3^ The recent paper by Long et al.,^4^ OA global trends boosted by over 113.25 %, with an increase of 247.51 million in 1990 to 527.81 million in 2019. It is estimated that over 240 million people suffer from symptomatic OA worldwide, 10 % of men and 18 % of women above 65 years.^5^^,^^6^ The knee represents the main joint between the four OA sites,^4^^,^^7^, ^8^, ^9^ significantly reducing the patient's mobility and independence.^10^, ^11^, ^12^, ^13^ One of the most important goals of orthopedic surgeons in knee osteoarthritis (KOA) treatment is to repair cartilage and restore articular homeostasis.^14^, ^15^, ^16^, ^17^ Stem cell therapy represents a viable and innovative conservative treatment option. Stem cells can evolve into different cell lines, and they can be classified into three types: embryonic-derived stem cells (ESCs), adult stem cells (ASCs), and induced stem cells (iPSCs)^18^^,^^19^ In particular, the research focuses on mesenchymal stem cells (MSCs), a type of ASCs that develop from the mesodermal layer and progressor tissues such as chondrocytes, adipocytes, and bone cells. Attention was concentrated after several studies that analyzed how these MSC cells in vitro could differentiate into chondrocytes and stimulate the proliferation of local progenitor cells of the joint.^20^, ^21^, ^22^ It is estimated that MSCs create a microenvironment of cytokines and growth factors that stimulate cartilaginous tissue regeneration and the upregulation of senescent chondrocytes that are still metabolically active, allowing for repair.^23^ ASCs are readily isolated from different tissues within the body,^24^ and the most used and successful methodology worldwide is autologous stem cells derived from adipose tissue.^25^^,^^26^ The best terminology subdivision considers the harvesting technique and dividing therapies based on adipose tissue stem cells into three types.^27^ Microfragmented Adipose Tissue (MAT) technique, which represents a type of autologous adipose-derived MSC in which the adipose tissue is separated mechanically without the use of enzymes; the fat is micro-fragmented. This method involves minimal manipulation of the adipose tissue to preserve the natural extracellular matrix, potentially enhancing the viability and efficacy of the stem cells. The Stromal Vascular Fraction (SVF) harvesting technique, that is obtained through enzymatic digestion of adipose tissue, followed by centrifugation to isolate a heterogeneous cell population that includes MSCs from the matrix.^27^^,^^28^ This method provides a rich source of stem cells, which can be used immediately without expanding in culture. Even though they are like SVF, MAT has been shown to have a different biological composition and constitute a completely different product.^29^ Another harvesting technique is Adipose-Derived Stem Cell (ADSC), with subsequent expansion of the cells in culture. This approach allows for the cultivation and proliferation of a more homogeneous population of stem cells, potentially increasing their therapeutic efficacy.^12^ Many studies were conducted to show the effectiveness of these therapies, both in vitro and *in vivo*, and several systematic reviews have been conducted. Still, none have analyzed only the cohort of patients with an average age over 65.^1^^,^^13^^,^^30^, ^31^, ^32^ This data is crucial because KOA although multifactorial, KOA is strongly age-related and mainly affects the population over 65. Our research started from this point, and the aims are 1) to evaluate the effectiveness of ADSC therapies in treating KOA in patients over 65 years old and 2) to evaluate the procedure's safety and assess the complication rate even in older people.

## 2 Materials and methods

In this systematic review, studies that compared postoperative outcomes and complications in patients who underwent knee injection of Adipose Stem Cells using different techniques were included. Our focus was on patients aged 65 years and older.

### 2.1 Inclusion and exclusion criteria

In this systematic review, we included prospective and retrospective studies that evaluated the outcomes and the complications of the intra-articular injection of ADSCs. We included studies that reported patients mean age over 65 years old and diagnosis of idiopathic or secondary grade II or III knee OA evaluated with Kellgren– Lawrence (KL) OA classification in multiple compartments (medial or lateral tibiofemoral joint or the patellofemoral), who received treatment with intra-articular injection of adipose tissue-derived mesenchymal stem cells (ADSCs). We excluded studies involving patients mean age under 65 years old and with other knee pain diagnoses; case reports, case series, and reviews were also excluded.

### 2.2 Search methods

A systematic literature search was performed using the following databases: Pubmed, Scopus, and Cochrane Library. We used the following search strategy: ("adipose"[All Fields] OR "adiposities"[All Fields] OR "adiposity"[MeSH Terms] OR "adiposity"[All Fields]) AND ("analogs and derivatives"[MeSH Subheading] OR ("analogs"[All Fields] AND "derivatives"[All Fields]) OR "analogs and derivatives"[All Fields] OR "derivatives"[All Fields] OR "derivable"[All Fields] OR "derivant"[All Fields] OR "derivants"[All Fields] OR "derivate"[All Fields] OR "derivated"[All Fields] OR "derivates"[All Fields] OR "derivation"[All Fields] OR "derivations"[All Fields] OR "derivative"[All Fields] OR "derive"[All Fields] OR "derived"[All Fields] OR "derives"[All Fields] OR "deriving"[All Fields]) AND ("stem cells"[MeSH Terms] OR ("stem"[All Fields] AND "cells"[All Fields]) OR "stem cells"[All Fields] OR ("stem"[All Fields] AND "cell"[All Fields]) OR "stem cell"[All Fields]) AND ("knee"[MeSH Terms] OR "knee"[All Fields] OR "knee joint"[MeSH Terms] OR ("knee"[All Fields] AND "joint"[All Fields]) OR "knee joint"[All Fields]). The research was executed on Feb 7, 2024. The articles' reference lists underwent a manual screening process. After duplicates were eliminated, two review authors (F.R.P. and G.F.P.) independently examined the abstracts of all relevant papers. Any uncertainties or conflicts were reviewed with the third reviewer to eliminate potential inaccuracies. Two reviewers (F.R.P and G.F.P.) evaluated the articles to identify which should be included in the review.

### 2.3 Data collection, analysis, and outcomes

Data extraction was independently produced by two reviewers (A.Z. and G.F.P.). We extracted the following study characteristics: authors, year of publication, type of study, number of patients, number of knees, sex, Kellgren-Lawrence classification, culture ADSC, Number of cells injected, mean follow-up, adverse events, significant complications, knee pain, swelling, local heat, stiffness, effusion. Finally, outcomes included clinical scores such as Visual analog scale (VAS), Knee injury and Osteoarthritis Outcome score (KOOS), International Knee Society score (IKS), Western Ontario and McMaster Universities Osteoarthritis Index (WOMAC), and Lysholm knee score.

### 2.4 Risk of bias assessment

The risk of bias for included studies was assessed by 2 independent reviewers (F.R.P. and G.F.P.) using the Methodological Index for Non-Randomized Studies (MINORS) score.

## 3 Results

### 3.1 Electronic search

**Fig. 1:**
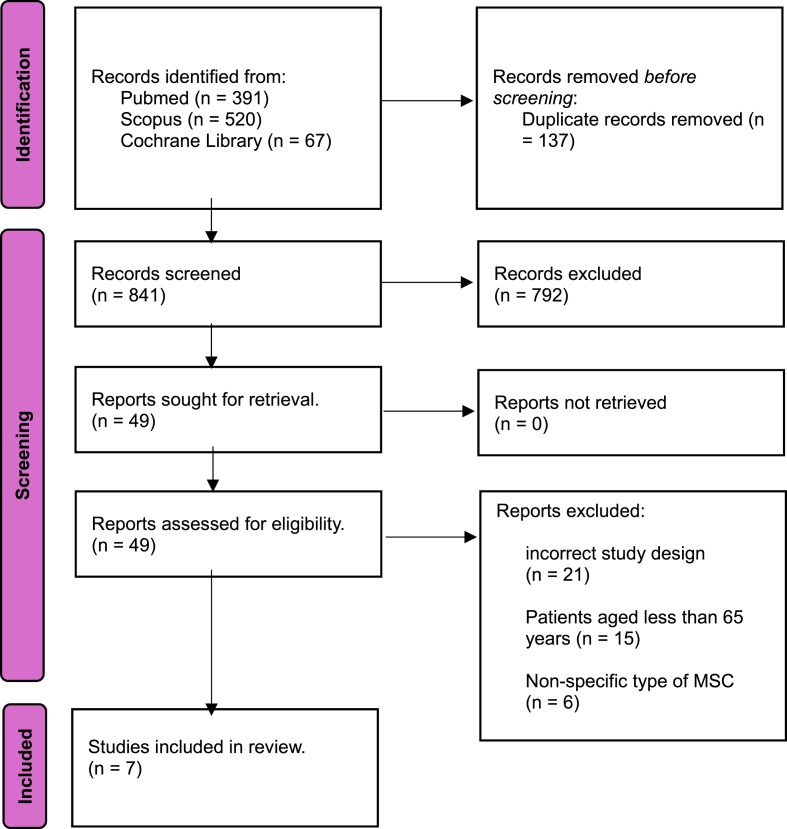
PRISMA 2020 flow diagram for new systematic reviews, which included searches of databases and registers only.

The literature search identified 978 articles. Of these, 841 articles were screened based on title and abstract after 137 duplicates were removed. Then 49 articles were read in full text, and 42 of these were excluded for the following reasons: incorrect study design (n = 21), patients aged less than 65 years (n = 15), and non-specific type of MSC (n = 6). Finally, we included seven articles in this review according to PRISMA^33^ (Fig. 1).

### 3.2 Demographic data

The overall number of participants in all the studies was 303 patients. The number of knees included in the studies was 339. The mean age of the participants was 69.5 years (65–82) (Table 1). The mean follow-up was 12.95 months.

**Table 1:** Demographic characteristics of included studies.

Author (year)	Type of Study	LOE	Study group	N patients	N knee	Age	Sex		K-L classification				Number of cells injected
							Male	Female	I	II	III	IV	
Higuchi et al. (2020)	RPS	III	ADSC	34	57	67.5 ± 11.1	10	24					8.26 ± 4.62 × 10^7
Koh et al. (2013)	CS	IV	ADSC	30	30	70.3	5	25					4.2 x 10^7
Schiavone Panni et al. (2018)	RPS	III	ADSC	52	52	67.3	22	32	11	19	22		N.R.
Tsubosaka et al. (2020)	PCS	IV	ADSC	57	57	69.4 ± 6.9	41	16		11	36	10	2.5 x 10^7
Chen et al. (2021)	RPS	III	ADSC	57	57	67.6 ± 6.60	11	46		37	37		N.R.
Yokota et al. (2017)	PS	II	ADSC	13	26	74.5 ± 5.4	2	11			2	11	3 x 10^7
Tsubosaka et al. (2021)	PS	II	ADSC LOW	30	30	69.0 ± 8.3	19	11		4	15	11	4.2 + 1.8 x 10^7
			ADSC HIGH	30	30	70.7 ± 5.3	24	6		5	17	8	8.5 + 3.1 x 10^7

LOE: Level of Evidence; ADSC: Adipose-Derived Stem Cells; K-L: Kellgren-Lawrence; N.R.: not reported; RPS: retrospective study; PCS: prospective case series; PS prospective studies; CS: case series.

### 3.3 Procedural and harvesting techniques data

Among the studies included in our review, various procedural and harvesting techniques were employed, reflecting the diverse approaches to MSC therapy. Only 1 study employed the MAT technique, 3 used the SVF harvesting technique, and 3 used the ADSC harvesting technique, with subsequent expansion of the cells in culture. The analysis of cellularity, which refers to the number of cells present in the final injected product, was reported by five out of seven studies. The mean number of cells injected across these studies was 5.11 × 10^7^. However, the volume of the cellular product was reported in only 2 studies. Notably, the first one of these studies did not report the cellularity of the injected product. Instead, a preformed kit from a commercial company was utilized to prepare the injection, highlighting a different approach in standardizing the procedure. The second one reported the volume involved a bilateral injection of 2.5 ml of the product, which was prepared using the SVF technique. This detail underscores the variability in the preparation and administration protocols across different studies. All studies consistently reported that adipose tissue was harvested from the abdominal area. This commonality suggests a preference for this site due to its accessibility and the abundant adipose tissue available. Regarding the infiltration techniques, two studies performed the injections using an ultrasound-guided procedure. In one study the infiltration was performed through an arthroscopic portal at the end of a microfracture procedure and only after the complete removal of the arthroscopic solution. The infiltration technique was not specified in the remaining studies, which leaves some uncertainty about the procedural consistency across these trials. Furthermore, only two studies considered multiple cohorts of patients who received different cellular concentrations of the product. This variation in dosage highlights the need for further research to determine the optimal cell concentration for therapeutic efficacy.

### 3.4 Outcomes and complications

Six papers mentioned the VAS score as the most frequent primary outcome. The mean value of the VAS score improved from 5.91 before the procedure to 3.62 at the final follow-up. Two papers reported an increase in KOOS scores. One paper mentioned WOMAC, IKS, and Lysholm knee scores. All the included studies improved all outcomes scores (Table 2). It is important to note that in two of these studies, bilateral knee injections were performed. However, these studies did not specify whether the clinical scores were evaluated separately for each knee or assessed collectively. This lack of distinction could impact the interpretation of the results, as individual knee responses may vary and affect the overall outcomes reported. Of 339 knees, 43 patients reported adverse events without significant complications, including knee pain, femoral local heat, swelling, effusion, and stiffness. These complications completely improved in all patients within a few days to 1 week. No major complications were reported. In the other studies, the most frequent early complication was knee pains, reported in 5 studies with a percentage of (6,2 %), followed by swelling reported in 2 studies with a percentage of 2,65 % (Table 3).

**Table 2:** Preoperative and postoperative clinical outcomes.

Author (year)	Follow Up	VAS pre	VAS 1M	VAS 3M	VAS 6M	VAS 12M	KOOS pre	KOOS 1M	KOOS 3M	KOOS 6M	KOOS 12M
Higuchi et al. (2020)	6 M	6.5 ± 2.5			3.5 ± 2.9		54.4 ± 12.7			64.6 ± 13.8	
Koh et al. (2013)	16.3 M	4.7 ± 1.6				1.7 ± 1.4					
Schiavone Panni et al. (2018)	15.3 M	8.5				5.1					
Tsubosaka et al. (2020)	12M	4.7				3.3					
Chen et al. (2021)	24M										
Yokota et al. (2017)	6 M	7.3 ± 1.8	4.9 ± 2.1		4.3 ± 2.4						
Tsubosaka et al. (2021)	12M	5.3	3.5	3.2	3	3	90	100	102	110	102
	12M	4.5	3.0	2.8	3	4	100	110	115	110	115

**Table 3:** Complications.

Author (year)	Infections	Knee pain	Swelling	Effusion	Local heat	Stiffness
Higuchi et al. (2020)	0	5 (9.6 %)	0	6 (11.5 %)	1 (1.9 %)	1 (1.9 %)
Koh et al. (2013)	0	3 (10 %)	0	0	0	0
Schiavone Panni et al. (2018)	0	2 (3.84 %)	0	0	0	0
Tsubosaka et al. (2020)	0	0	0	0	0	0
Chen et al. (2021)	0	9 (15 %)	6 (10.5 %)	1 (1.75 %)	1 (1.75 %)	3 (5.26 %)
Yokota et al. (2017)	0	0	0	0	0	0
Tsubosaka et al. (2021)	0	2 (3.3 %)	3 (5 %)	0	0	0

### 3.5 Evidence of the included studies

Higuchi et al.^34^ analyzed 34 patients (57 knees); 23 patients received a bilateral injection. The mean age of 67.5 years, the number of cells injected was 8.26 ± 1.62 × 10^7^. The study showed an improvement in VAS score from 6.5 ± 2.5 to 3.5 ± 2.9 and a KOOS score of 54.4 ± 12.7 to 64.6 ± 13.8 at the final follow-up of 6 months. During the observation period, there were no significant adverse events or problems. All the patients' symptoms, including 1 case of stiffness, 1 case of local heat, 5 cases of knee pain, and 6 cases of effusion, transient and completely improved in all patients within 7 days. Koh et al.^35^ analyzed 30 patients (30 knees) with a mean age of 70.3 years, the mean cellularity was 4.2 × 10^7^ cells injected and showed an improvement of VAS score from 4.7 ± 1.6 to 1.7 ± 1.4 and Lysholm from 54.3 ± 15.4 to 74.2 ± 13.4 at final follow-up of 16.3 months. They reported only 3 cases of knee pain solved in 10 days and non-major complications and infections. Schiavone Panni et al.^36^ examined 52 patients (52 knees) with a mean age of 67.3 years and demonstrated an improvement of VAS score from 8.5 to 5.1 and IKS from 37.4 to 62.4 at the final follow-up of 15.3 months. They reported only 2 cases of knee pain resolved in a few days. Tsubosaka et al.^37^ analyzed 57 patients (57 knee) with a mean age of 69.4 years, mean of injected cells of 2.5 × 10^7^ and showed an improvement in VAS score from 4.7 to 3.3 and WOMAC score from 33.4 to 22.6. at the final follow-up of 12 months. There were no serious adverse events or complications during the observation period. Chen et al.^38^ examined 57 patients (57 knees) with a mean age of 67.6, and they reported in injection site condition, 9 cases of knee pain, 6 cases of swelling, 1 of effusion and local heat, and 3 of stiffness. These problems were completely improved in all patients without sequel. Yokota et al.^39^ analyzed 13 patients (26 knees), all received a bilateral injection and the mean cellularity was 3 × 10^7^ with a mean age of 74.5 years. They showed an improvement in VAS score from 7.3 ± 1.8 to 4.9 ± 2.1 at 1-month follow-up and 4.3 ± 2.4 at final follow-up of 6 months. During the observation period, there were no significant adverse events or problems. The most recent paper by Tsubosaka et al.^40^ analyzed 60 patients divided into two groups, a total of 30 patients were assigned to the high-dose group, with an intra-articular injection of 8.5 × 10^7^ SVF cells with a mean age of 69 years, whereas the remaining 30 patients received an intra-articular injection of 4.2 × 10^7^ SVF cells (low-dose group) with a mean age of 70.7 years. The high-dose group showed an improvement in VAS score from 4.5 to 3 and KOOS from 100 to 115 at the final follow-up of 12 months, whereas the low-dose group reported an improvement of VAS score from 5.3 to 4 and KOOS from 90 to 102 at the final follow-up of 12 months. They reported only 2 cases of knee pain solved in 3 days and no severe complications.

### 3.6 Quality assessment

The quality of included studies, assessed by MINORS, obtained a mean value of 13, ranging from 10 to 15 (Table 4).

**Table 4:** MINORS.

Author	Stated aim	Inclusion of patients	Collection of data	Endpoints appropriate to the aim	Unbiased assessment of the study endpoint	Follow-up	Loss to follow up less than 5 %	Prospective calculation of the study size	Total
Higuchi et al. (2020)	2	2	2	2	0	1	2	1	12
Koh et al. (2013)	2	2	2	2	1	2	2	1	14
Schiavone Panni et al. (2018)	2	2	2	2	0	2	2	1	13
Tsubosaka et al. (2020)	2	2	2	2	0	2	2	1	13
Chen et al. (2021)	2	2	2	2	2	2	2	1	15
Yokota et al. (2017)	2	2	1	2	0	1	2	0	10
Tsubosaka et al. (2021)	2	2	2	2	0	2	2	1	13

## 4 Discussion

The current systematic review improved the latest research and showed findings on the potential of adipose-based therapies in treating knee OA. Therapies with ADSC have been evaluated as effective in the elderly over 65, with significant results regarding clinical outcomes. The literature analysis leads to encouraging assessments for applying these innovative therapies. OA is improving, as is the average age of active elderly patients. The analyses reported allow us to consider ADSC procedures as a therapeutic alternative also in the elderly population. Our findings align with most of the literature that analyzed MSC therapies' effects. In one of the last reviews, Shimozono et al.^13^ focused their research on describing and classifying all MSC techniques. They expressed in detail the scientific and clinical evidence present in the literature for each single procedure. This study showed that ADSCs expanded in culture and presented encouraging results but were limited by costs. However, all MSC procedures had a differentiating effect at the joint level, particularly for immunomodulatory and microenvironmental changing action. A further review with positive results is the study by Hurley et al.,^31^ which analyzed the scientific production in the literature regarding the effects and safety of ADSCs in the form of SVF. Their study focused on studying the effectiveness of the therapy in treating all forms of OA and not specifically KOA. However, treatment demonstrated effectiveness and safety but with limitations, considering several studies with biological adjuvants, which have inevitably confused the results. Another critical review by Filardo et al..^32^ group concluded by clearly expressing how, in 2012, it was difficult to highlight reliable results due to excessive confusion in the techniques described in the literature and the indication for the use of ADSC procedures. More recently, the review by Buzaboon et al.^1^ in 2020 analyzed the clinical effectiveness and safety of these biological therapies by taking MSCs into broader consideration without making a distinction between the tissue from which they were taken. This review demonstrated that mesenchymal stem cells were more effective in treating OA than conventional treatments, significantly improving all clinical outcomes (KOOS, VAS, WOMAC). All reviews analyzed studies with patients with an average age of less than 65 years. According to World Health Organization (WHO) guidelines, elderly patients are over 65, and our review focused on analyzing only articles that studied the effectiveness of ADSC therapies in elderly patients. Regarding age, the results regarding the safety of these innovative procedures were critical, which were no less important. The elderly population is made up mostly of frail people with multiple chronic pathologies and pharmacological therapies. The risk analysis of complications was therefore necessary. Data from the current review showed that the risk of complications was not higher in the elderly. This finding encourages orthopedic surgeons not to consider surgery as the only solution in elderly who are refractory to treatment and do not show end-stage KOA. Surgery allows for the restoration of anatomy and joint function but only in end-stage osteoarthritis, and it carries several risks and complications.^41^ The results of this systematic review, which was analyzed individually, align with those obtained from the studies considering younger cohorts of patients. Among these relevant studies are Lu et al.,^42^ Lee et al.,^43^ and Freitag et al.,^24^ who conducted comparative studies comparing the joint injection of ADSC with hyaluronic acid or saline solution, showing an improvement in clinical outcomes at 12 months in the group subjected to infiltration of fat stem cells. In particular, the working group of Lee and Lu, together with that of Zhang et al.,^5^ also performed an MRI evaluation and demonstrated a notable increase in cartilage volume in patients treated with stem cells. These results agree with Tsubosaka et al.^40^ and Higuchi et al.,^34^ highlighting an improvement in the cartilage framework through MRI thanks to the MOAKS classification. A fascinating study by Kim et al.^44^ conducted a 5-year follow-up and analyzed how a single ADSC injection performed at high doses of ADSC leads to a clinical improvement with a low-risk profile without worsening the radiological picture after five years. Kim's study suggested that ADSC-based therapies could be a treatment that modified the natural history of KOA. It also describes the increasingly frequent use of stem cell therapies with conventional arthroscopic joint cleaning and washing procedures. One comparative studies of this type were included in the current review, but significant on this topic are the comparative studies by Koh et al.,^35^ Kim et al.,^44^ and Vasso et al.,^45^ who compared the results of arthroscopy without and with injection of SVF showing an improvement in both clinical and radiological outcomes in all cases. Regarding the effectiveness and safety of the application of MAT, several studies have been conducted; relevant are those of the Italian group of Onorato et al.^46^ and Screpis et al.,^47^ who showed how this procedure is safe and effective with a significant improvement in knee clinical scores in early and moderate OA. An important finding regarding clarifying clinical scores is that the analysis did not specify which knee was evaluated. This represents a significant limitation, especially when using scores like KOOS, Lysholm, WOMAC or all scores, because clinical results can influence the performance, potentially skewing the interpretation of outcomes. The presence of studies reporting ultrasound-guided injections is an exciting finding but should be interpreted with caution. According to the literature, ultrasound guidance can potentially enhance the accuracy of injections by providing real-time visualization of the target area.^48^, ^49^, ^50^, ^51^ These studies showed that in-plane ultrasound-guided knee injections for the lateral suprapatellar approach are safe and effective compared to landmark palpation-guided techniques. These studies are relevant as ultrasound-guided arthrocentesis and injections significantly reduce pain, increase success rates and improve clinical outcomes. However, it is essential to note that the necessity of ultrasound guidance for ensuring greater precision in knee injections remains a topic of debate. While it may reduce the risk of extra-articular (failed) injections, which can cause pain and swelling, no consistent data supports the mandatory use of ultrasound guidance in all cases.^52^^,^^53^ Further research is needed to establish clear guidelines on using ultrasound guidance to optimise the outcomes of knee injections and minimise complications. In summary, these insights highlight the need for careful consideration of procedural details and outcome assessments in future studies to improve the standardisation and reliability of MSC therapies for knee treatments. The results of this systematic review, as well as all previous ones and the studies cited above, described how autologous adipose-derived stem cells are an effective and safe procedure and valid therapy to address KOA. This review has limitations already found in previous cases, but the most critical limit is related to the quality and type of studies, which are very heterogeneous. A meta-analysis was not performed for heterogeneity across the studies regarding dosing regimens, patient selection, and outcome measures. These differences prevented meaningful pooling of data. The primary sources of heterogeneity included variations in the number of cells injected, frequency of injections, treatment protocols, patient demographics, clinical profiles, and assessment tools. Additionally, the adipose tissue is susceptible to anthropometric parameters and hormonal or pharmacological therapies that affect its biological and functional profile. In vivo studies are therefore necessary to evaluate any changes in the performance of adipose cells due to these considerations. As explained by Ossendorf et al.,^27^ the differences in the application of MSC-based therapies worldwide and regulatory and economic differences limit the use and, consequently, the study of all treatments. To address these limitations, future research should aim for greater standardisation of experimental protocols regarding using Mesenchymal Stem Cells (MSCs) in knee treatments. Harmonising dosing regimens, establishing uniform criteria for patient selection, and adopting consistent outcome measures will enhance the comparability of studies. This standardisation will facilitate more robust meta-analyses and the synthesis of scientific evidence, advancing clinical practice and improving patient outcomes. Researchers can contribute to a clearer understanding of MSC treatment efficacy and safety by striving for greater methodological consistency.

## 5 Conclusion

This systematic review examined the current literature on autologous adipose-derived stem cell procedures in elderly people. Evaluating clinical outcomes in the treatment of OA in the elderly, we demonstrated that all procedures had favorable clinical outcomes with minimal risk also in this population.

## Statements and declarations

The authors declare that no funds, grants, or other support were received during the preparation of this manuscript.

The authors have no relevant financial or non-financial interests to disclose.

All authors contributed to the study's conception and design, and all authors read and approved the final manuscript.

Availability of data and material, code availability are not applicable.

The authors grant permission for the publication of the study.

Informed consent is not applicable.

## Credit author statement

**Conceptualisation**: Biagio Zampogna:

**Methodology, Software**: Giuseppe Francesco Papalia, Parisi Francesco Rosario, Saseendar Shanmugasundaram.

**Data curation**: Parisi Francesco Rosario, Andrea Zampoli, Augusto Ferrini.

**Writing- Original draft preparation**: Biagio Zampogna, Parisi Francesco Rosario, Andrea Zampoli.

**Visualization, Investigation**: Andrea Zampoli, BIagio Zampogna, Parisi Francesco Rosario.

**Supervision**: Rocco Papalia, Giuseppe Francesco Papalia, Saseendar Shanmugasundaram.

**Software, Validation**: Giuseppe Francesco Papalia, Francesco Rosario Parisi.

**Writing- Reviewing and Editin**: Biagio Zampogna, Rocco Papalia, Saseendar Shanmugasundaram.

## Declaration of competing interest

The authors declare that they have no known competing financial interests or personal relationships that could have appeared to influence the work reported in this paper.
